# Breast cancer cell‐derived exosomal miR‐20a‐5p promotes the proliferation and differentiation of osteoclasts by targeting SRCIN1

**DOI:** 10.1002/cam4.2454

**Published:** 2019-08-06

**Authors:** Ling Guo, Ye Zhu, Liandi Li, Shufen Zhou, Guohua Yin, Guanghao Yu, Hujun Cui

**Affiliations:** ^1^ Department of Pathology The Affiliated Second Hospital Mudanjiang Medical University Mudanjiang Heilongjiang China; ^2^ Department of Obstetrics Gynecology Peking University People's Hospital Beijing China; ^3^ Department of Anesthesiology The Affiliated Second Hospital Mudanjiang Medical University Mudanjiang Heilongjiang China; ^4^ Department of Gerontology The Affiliated Second Hospital Mudanjiang Medical University Mudanjiang Heilongjiang China; ^5^ Department of Nursing The Affiliated Second Hospital Mudanjiang Medical University Mudanjiang Heilongjiang China; ^6^ Department of Medical Imaging Mudanjiang Medical University Mudanjiang Heilongjiang China; ^7^ Department of Oncology The Affiliated Hongqi Hospital, Mudanjiang Medical University Mudanjiang Heilongjiang China

**Keywords:** bone metastasis, breast cancer, exosomes, miR‐20a‐5p, SRCIN1

## Abstract

Bone metastasis of breast cancer makes patients suffer from pain, fractures, spinal cord compression, and hypercalcemia, and is almost incurable. Although the mechanisms of bone metastasis in breast cancers have been studied intensively, novel specific target will be helpful to the development of new therapeutic strategy of breast cancer. Herein, we focused on the microRNA of tumor cell‐derived exosomes to investigate the communication between the bone microenvironment and tumor cells. The expression of miR‐20a‐5p in the primary murine bone marrow macrophages (BMMs), MCF‐10A, MCF‐7, and MDA‐MB‐231 cell lines, as well as the cell‐derived exosomes were assessed by qRT‐PCR. Transwell assays were used to evaluate the effects of miR‐20a‐5p on tumor cell migration and invasion. The expression of exosomes marker including CD63and TSG101 was detected by Western Blot. Cell cycle distribution of BMMs was analyzed by flow cytometry. 3‐UTR luciferase reporter assays were used to validate the putative binding between miR‐20a‐5p and SRCIN1. MiR‐20a‐5p was highly expressed in breast tumor tissues and the exosomes of MDA‐MB‐231 cells. MiR‐20a‐5p promoted migration and invasion in MDA‐MB‐231 cells, and the proliferation and differentiation of osteoclasts. MDA‐MB‐231 cell‐derived exosomes transferred miR‐20a‐5p to BMMs and facilitated the osteoclastogenesis via targeting SRCIN1. The present work provides evidence that miR‐20a‐5p transferred from breast cancer cell‐derived exosomes promotes the proliferation and differentiation of osteoclasts by targeting SRCIN1, providing scientific foundations for the development of exosome or miR‐20a‐5p targeted therapeutic intervention in breast cancer progression.

## Graphical abstract

Highlight：(a) MiR‐20a‐5p promotes migration and invasion in MDA‐MB‐231 cells. (b) MDA‐MB‐231 cell‐derived exosomes transferred miR‐20a‐5p to BMMs and facilitated the osteoclastogenesis via targeting SRCIN1. (c) The study provides evidence that miR‐20a‐5p transferred from breast cancer cell‐derived exosomes promotes the proliferation and differentiation of osteoclasts by targeting SRCIN1.

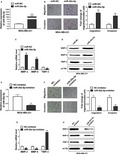

## 1 INTRODUCTION

Breast cancer is a worldwide crucial public health problem, which is the second leading cause of malignant death among women, representing approximately one third of diagnosed cancer among women in the United States.^1^ According to recent epidemiology data, relative survival rate at 5 years for women diagnosed with breast cancer is 91%,^2^ where metastasis accounts for 90% of deaths, with a very limited survival.^3^ As high as 70% of breast cancer patients develop bone metastasis during the disease progression.^4^ In addition, skeleton is also the first site of metastasis for about 26%‐50% of patients with metastatic breast cancer.^5^ While early detected in situ ductal carcinoma is 98% curable, bone metastasis is basically incurable,^6^ accompanying bone pain, bone fractures, hypercalcemia, and spinal cord compression.^7^ Osteolytic lesions are found in as high as 80% of patients with stage IV metastatic breast cancer,^8^ characterized by the net bone destruction and increased osteoclast activity.^9^ The mechanisms of bone metastasis in breast cancers have been studied intensively. Malignant bone lesions are classified by their osteolytic and osteoblastic extents radiographically.^10^ Four types of cells are involved in bone metastasis establishment: tumor cells, osteoblasts, osteoclasts, and mineralized bone matrix.^7^ The bone matrix is the substantial source of the growth factors, such as transforming growth factor‐β (TGF‐β), fibroblast growth factor, platelet‐derived growth factor, insulin‐like growth factors, and bone morphogenetic proteins, which are released by osteolysis and further promote the proliferation of tumor cells.^11^ Moreover, the physical features of the bone matrix, including hypoxia, acidosis, and aberrant extracellular calcium concentration, codriving a favorable environment for tumor proliferation.^12^ In general, the communication between the bone microenvironment and tumor cells promotes a feedback loop of bone metastasis. Deeper understanding of the interaction between bone environment and tumor cells may result in the identification of potential targets, and the development of emerging chemotherapeutic intervention towards bone metastasis in breast cancer.

The secretion of extracellular vehicles (EVs) serves one communicates way between cells and neighboring or distant cells. EVs are composed of transmembrane proteins and enclosing cytosolic proteins and RNA. Exosomes are small endocytic EVs (30‐100 nm) secreted by various cell types such as dendritic cells, melanoma cells, microglia, mast cells, colorectal cancer cells, hepatocytes, breast cancer cells etc.^13^ Exosomes mediates diverse biological functions including cell‐cell communication, tumor cell invasion, and antigen presentation through mRNAs, microRNAs (miRNA), and protein transfer.^14^ Recent evidence suggests that cancer cell‐derived miRNAs can be transferred via exosomes to endothelial cells to promote angiogenic effects.^15^ In addition, miR‐10b, one exosomal miRNA secreted by breast cancer cells, has been reported to impact tumor development and progression.^14^ However, whether and how exosomes participate in cell invasion to skeleton remains poorly understood. Therefore, the aim of this study was to identify the role of exosomes, particularly miR‐20a‐5p, in breast cancer invasion.

## 2 MATERIALS AND METHODS

### 2.1 Clinical specimens

Human breast cancer tissue samples and adjacent normal mammary tissues were obtained from patients diagnosed at the Affiliated Hongqi Hospital, Mudanjiang Medical University, according to the clinical human specimen protocol approved by the Institutional Research Ethics Committee in the Affiliated Hongqi Hospital, Mudanjiang Medical University (#HQH6304AQ2). Patients’ written consents were obtained prior to the surgery. All specimens were frozen in liquid nitrogen immediately and stored at −80°C for subsequent miRNA and gene quantitation assays.

### 2.2 Human cell line culture

Normal human mammary MCF‐10A cell line, human breast cancer cell lines including MCF‐7 and MDA‐MB‐231 were purchased from the American Type Culture Collection (ATCC). Cells were grown in Dulbecco's modified Eagle's medium (DMEM), supplemented with 10% fetal bovine serum (FBS) and G418 (Thermo Fisher Scientific), and maintained at 37°C in a humidified cell incubator with 5% (v/v) CO_2_.

### 2.3 Mouse bone marrow‐derived macrophages culture

C57BL/6 mice (6‐8 weeks old) were purchased from Shanghai Laboratory Animal Center. All animal experiments were performed in accordance with the animal protocol approved by the Institutional Animal Care and Use Committee of the Affiliated Hongqi Hospital, Mudanjiang Medical University. For bone marrow cells preparation, mice were sacrificed by cervical dislocation, followed by the removal of femurs and tibias. Prechilled PBS containing 2% FBS was used to flush the bone marrow by syringe with 25‐gauge needle. The red blood cells in the cell suspension were lysed using M‐lysis buffer (R&D Systems). Furthermore, bone marrow cells were cultured in alpha‐minimum essential medium (MEM) with 10% FBS in cell incubator at 37°C. After 3 days of incubation, the supernatant was discarded, and the adherent cells were obtained as BMMs for subsequent experiments.

### 2.4 Exosome isolation and quantitative real time PCR

To isolate exosomes from cells, the conditioned medium culturing these two cell lines (MCF‐10A exo and MDA‐MB‐231 exo) after 48 hours incubation, were collected and centrifuged at 1500 *g* for 15 minutes at 4°C. After filtering through a 0.22 mm filter (Millipore‐Sigma), the conditioned medium was ultracentrifuged twice at 110 000 *g* for 1 hour at 4°C, and the pellets were resuspended in PBS. The isolated exosomes were identified under electron microscopy to observe the morphology and size.

Total miRNAs from the tissue samples, cultured cells and the isolated medium were extracted with the miRNAprep Pure FFPE Kit (Tiangen Biotech) according to the manufacturer's instruction. cDNA was synthesized using the Taqman miRNA reverse transcription kit (ThermoFisher Scientific). qRT‐PCR was performed to amplify the cDNA templates by. Quantitative real‐time PCR was performed on a CFX‐1000 real‐time PCR system (Bio‐Rad). The relative mRNA expression levels were calculated by the 2^−△△^Ct method and normalized to U6. After we got the expression level of miR‐20a‐5p in the 50 breast cancer tissues by qRT‐PCR, we rank these values from smallest to biggest, according to the specific value distribution, we defined the first 20 as miR‐20a‐5p low expressed, and the last 30 as miR‐20a‐5p high expressed. The specific primer sequences used were as following: miR‐20a‐5p RT primer, GTCGTATCCAGTGCAGGGTCCGAGGTATTCGCACTGGATACGACCTACCT; U6 RT primer, GTCGTATCCAGTGCAGGGTCCGAGGTATTCGCACTGGATACGACAAAATATGGAA; miR‐20a‐5p ‐F: GCCCGCTAAAGTGCTTATAGTG, miR‐20a‐5p ‐R: GCTGTCAACGATACGCTACGT; U6‐ F: TGCGGGTGCTCGCTTCGGCAGC, U6‐R: GTGCAGGGTCCGAGGT. MMP‐2 F: CTCAGCGGCTCATGGTCCGGCC; R: CATGGTCCGGCCCCCGCCCCCA. MMP‐9 F: ATTTCAGCCAAATAACTCACAT; R: TTCTTTCCCCACTTTACAAATGAGAAAAGG. TIMP3 F: CATGTGCAGTACATCCATACGG; R: CATCATAGACGCGACCTGTCA.

### 2.5 Western blot

Protein concentration was determined by Bradford protein assay, and total 15 µg of protein was loaded and separated by 10% SDS‐PAGE and transferred to nitrocellulose membrane (Bio‐Rad). After washing with 1xTBS buffer, the membranes were incubated with 5% nonfat milk in TBST (TBS, 0.1% Tween 20) for 2 hours for blocking. Subsequently, the membranes were incubated with primary antibody against CD63 (1:1000, Proteintech) and TSG101 (1:2000, Abcam) overnight at 4°C. Membranes were washed five times with TBST buffer, and incubated with the secondary antibodies for 1 hour at room temperature. The bands on the membrane were detected using enhanced chemiluminescence kit (Thermo Fisher Scientific).

### 2.6 In vitro cell migration and invasion assays

Cell migration assay was performed using Transwell chamber coated with Matrigel (BD Biosciences). Briefly, MDA‐MB‐231 cells were washed and resuspended with serum‐free DMEM as single cell suspension. Next, 100 μL of cell solution at 1.5 × 10^5^ cells/mL was plated on the top of the Transwell insert (8 μm pores in a 24‐well format) and incubated for 10 minutes at 37°C with 5% CO_2_. DMEM with 10% FBS was added to the lower basolateral chamber. After 10~12 hours incubation at 37°C, the chamber was removed, and cells that failed to penetrate through the membrane were rubbed away a P200 pipet tip. The remaining cells were then fixed with 95% ethanol for 15 minutes, and stained for 10 minutes by 0.1% crystal violet. After three times of PBS rinses and air‐drying, the chambers were inverted on a glass slide and photographed under microscope.

For cell invasion assay, firstly 50 μL of Matrigel diluted by serum‐free medium (1:7) was applied into each chamber. Likewise, 100 μL of MDA‐MB‐231 cell suspension was inoculated into the apical layer covered by diluted Matrigel, while 500 μL of culture medium with 10% FBS was added to the basolateral chamber. Cells invasion was monitored 10~12 hours later as described above using an inverted microscope.

MiR‐20a‐5p inhibitor was chemically synthesized by GenePharma (Shanghai, China) with the sequence of CTACCTGCACTATAAGCACTTTA.

### 2.7 Flow cytometry

Primary preosteoclasts were exposed to MCF‐10A cell‐derived exosomes (+MCF‐10A exo) or MDA‐MB‐231 cell‐derived exosomes (+MDA‐MB‐231 exo) or alone (blank). After 48 hours incubation, the cells were rinsed and resuspended with PBS to approximately 1 × 10^5^ cell/mL. Cells was fixed by prechilled 70% ethanol for 1 hour at 4°C, followed by the incubation with 100 μL of RNase A at 37°C for 30 minutes. Furthermore, 400 μL of propidium iodide (P4170, Sigma‐Aldrich) was added for 30 minutes staining. Cell cycles at G1, S, and G2 were analyzed by CytoFLEX Flow cytometer (Beckman Coulter) at a wavelength of 488 nm.

### 2.8 Cell viability assay

Primary preosteoclasts were incubated with MCF‐10A cell‐derived exosomes (+MCF‐10A exo) or MDA‐MB‐231 cell‐derived exosomes (+MDA‐MB‐231 exo) or alone (blank). After 24, 48, and 72 hours respectively, 20 μL of MTT (M2128, Sigma‐Aldrich) at 5 mg/mL was added to cell wells for 4 hours of additional culturing. The culture medium was discarded and 150 μL of Dimethyl sulfoxide (D5879, Sigma‐Aldrich) was added to each cell well. After overtaxing for 10 minutes, and the absorbance was measured at 570 nm. The relative cell viabilities in all groups were compared with the MDA‐MB‐231 exo group which showed the highest optical density.

### 2.9 TRAP staining

The cells in the above aforementioned designated groups were washed fixed in 4% paraformaldehyde, followed by the staining with Acid Phosphatase, Leukocyte (TRAP) Kit (387A, Sigma‐Aldrich) at 37°C for 30 minutes. The number of TRAP‐positive osteoclasts were observed and recorded under a microscope (DM1000, Leica).

### 2.10 Binding between SRCIN1 and miR‐20a‐5p

The target gene of miR‐20a‐5p was predicted using Targetscan (http://www.targetscan.org/vert_71). A target fragment of 3′‐UTR of SRCIN1 containing the putative miR‐20a‐5p binding site was amplified by PCR. The human SRCIN1‐WT 3′‐UTR plasmid was constructed by PCR amplification using primers as followed: F: CTACTCGAGAAGCCCCTCACCCCGCTG, R: CTAGCGGCCGC TCCAGGAGAGGAAAAAGAAACAA.

The human SRCIN1‐Mut 3′‐UTR plasmid was constructed by PCR amplification using primers as followed: F: GATTTAACCCCTGAAATGGCATTAAC, R: GTTAATGCCATTTCAGGGGTTAAATC.

The mouse SRCIN1‐WT 3′‐UTR plasmid was constructed using mmu‐SRCIN1 3′UTR primers: F: CTACTCGAGAAGCCCCTC ATGCCACCACCC, R: CTAGCGGCCGC TCCAGGAGAGAAAAAAGAAACAAGT.

The mouse SRCIN1‐Mut 3′‐UTR plasmid was constructed using mmu‐SRCIN1 3′UTR primers: F: AATTTACCCCGTCAAATGCCATTAA, R: TTAATGGCATTTGACGGGGTAAATT.

BMM and MDA‐MB‐231 cells were cultured on 96‐well plate and transfected with 100ng constructs with miR‐20a‐5p or miR‐NC (50 nmol/L per well) and SRCIN1 WT‐3′‐UTR or SRCIN1‐Mut 3′‐UTR (50 ng/well) using X‐tremeGENE Transfection Reagents (Roche). After 24 h, luciferase activities were measured using the dual luciferase assay kit (Promega).

In addition, to validate the role of SRCIN1 in the osteoclasts, SRCIN1 si‐RNA was transfected into BMMs, and the proliferation and differentiation of BMMs were detected using methods described above.

### 2.11 Pull‐down assay of target mRNAs of miR‐20a‐5p

The pull‐down assay of target mRNAs of miR‐20a‐5p was performed as described previously.^16^ Briefly, semiconfluent BMM and MDA‐MB‐231 cells on 90‐mm culture dishes were harvested and treated with 0.5 mL of lysis buffer, followed by the incubation with biotinylated double‐stranded RNA (8 nmoles) of miR‐20a‐5p. Furthermore, the extract was incubated with Streptavidin Mutein Matrix (Roche). The Streptavidin/biotin–miRNA/mRNA complex was collected and the relative enrichment of SRCIN1 was assessed by RT‐PCR using the following primers: mmu‐SRCIN1, F: AGCAGGACAGGATGCGAGAACA, R: TGATGAGGATGGCGGTGTTGG; has‐SRCIN1, F: GAACGGCTGCGCTATCTCAA, R: GGATCTTCTCCACCGATTTCTCC.

### 2.12 Statistical analysis

All data shown are expressed as mean ± standard deviation (SD) from at least three independent experiments. Student's *t* test, one‐ or two‐way ANOVA followed by a Tukey post hoc test was used to analyze the difference significance of between groups. Results with *P*‐value <.05 were considered statistically significant.

## 3 RESULTS

### 3.1 MiR‐20a‐5p was highly expressed in breast tumor tissues and the exosomes of MDA‐MB‐231 cells

**Figure 1:**
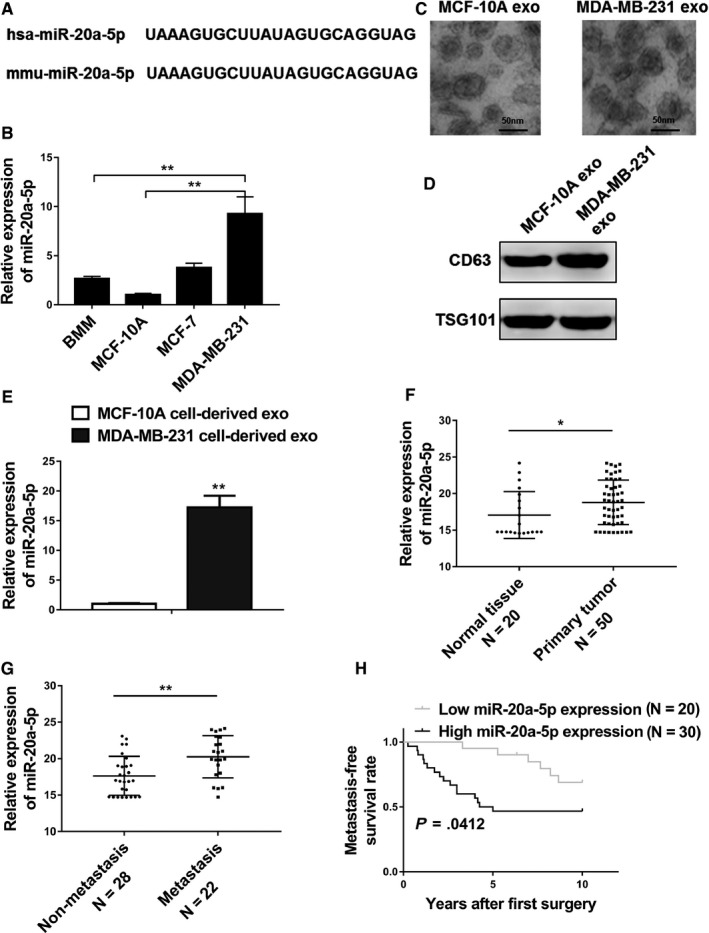
MiR‐20a‐5p was highly expressed in breast tumor tissues and the exosomes of MDA‐MB‐231 cells. A, A comparison of nucleotides of the mature miR‐20‐5p in humans and mice. B, qRT‐PCR analysis revealed miR‐20‐5p expression in primary murine bone marrow macrophages (BMMs), MCF‐10A cells, MCF‐7 cells, and MDA‐MB‐231 cells. C, Representative electron micrographs of exosomes isolated from MCF‐10A cell conditioned and MDA‐MB‐231 cell conditioned medium revealing the typical morphology and size (50‐150 nm), bar = 50 nm. D, Western blot analysis showing abundant CD63 and TSG101 in exosomes derived from the medium of MCF‐10A (MCF‐10A exo) and MDA‐MB‐231 (MDA‐MB‐231 exo) cells. E, qRT‐PCR analysis revealing miR‐20‐5p expression in MCF‐10A cell‐derived and MDA‐MB‐231 cell‐derived exosomes. F, Expression levels of miR‐20‐5p in 20 normal tissues and 50 breast cancer tissues. G, Relative expression levels of miR‐20‐5p in groups of breast cancer tissues classified based on the occurrence of bone metastasis (metastatic or nonmetastatic). H, Kaplan‐Meier's analysis of the correlation between miR‐20‐5p expression and the metastasis‐free survival of breast cancer patients. The data represent the mean ± SD from three independent experiments. *P < .05; **P < .01

As shown in Figure 1A, the sequences of hsa‐miR‐20a‐5p and mmu‐miR‐20a‐5p are identical. Therefore, we used the same primers in qRT‐PCR to detect the expression levels of miR‐20a‐5p in BMMs, MCF‐10A, MCF‐7, and MDA‐MB‐231 cells, respectively.

The results showed that MDA‐MB‐231 cell line had significant higher expression level of miR‐20a‐5p in comparison with those in other three cell groups (Figure 1B, *P* < .01). We further isolated exosomes secreted by the MCF‐10A and MDA‐MB‐231 cell lines by ultracentrifugation of the culture medium, and the electron micrographs of isolated exosomes demonstrated typical vesicles size 50‐150 nm (Figure 1C). The enrichment of exosomes was confirmed by Western blot analysis using the known markers CD63 and TSG101 (Figure 1D). Subsequently, we examined the expression levels of miR‐20a‐5p in the exosomes, and the results showed that MDA‐MB‐231 cell‐derived exosomes presented significantly upregulated miR‐20a‐5p (Figure 1E, *P* < .01). The quantitation of miR‐20a‐5p was also evaluated in the primary tumor tissue samples and adjacent normal tissues from breast cancer patients, and the data demonstrated significantly increased level of miR‐20a‐5p expression in tumor tissues (Figure 1F, *P* < .05). All of these results presented that miR‐20a‐5p was highly expressed in both cancer tissues and TNBC cell lines. Notably, we found patients with metastasis showed significant higher expression of miR‐20a‐5p compared to the nonmetastasis patients (Figure 1G), and patients with low miR‐20a‐5p level showed significant lower metastasis‐free survival rate compared to patients with high miR‐20a‐5p expression (Figure 1H), indicating the important role of miR‐20a‐5p in the metastasis and progression of breast cancer. In addition, individual analysis from 50 breast cancer patients showed no correlation between the expression of miR‐20a‐5p and the clinicopathological features such as age, tumor size, and marker status. However, patients with bone metastasis showed significantly higher expression of miR‐20a‐5p than patients without bone metastasis (*P* < .001, Table S1).

### 3.2 MiR‐20a‐5p promoted migration and invasion in MDA‐MB‐231 cells

**Figure 2:**
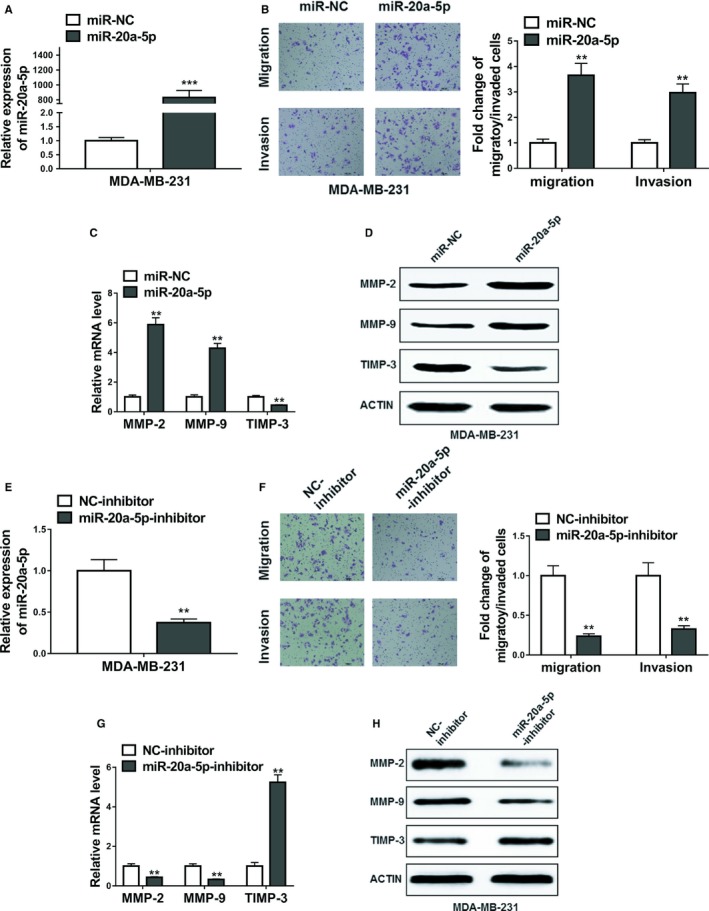
MiR‐20a‐5p promoted migration and invasion in MDA‐MB‐231 cells. A, The relative expression levels of miR‐20a‐5p in MDA‐MB‐231 cells transfected with miR‐20a‐5p mimics or miRNA negative control (miR‐NC). B, Transwell assay showed overexpression of miR‐20a‐5p promoted migratory and invasive abilities of MDA‐MB‐231 cells. C and D, The expression levels of MMP‐2, MMP‐9, and TIMP‐3 in MDA‐MB‐231 cells transfected with miR‐20a‐5p mimics or miRNA negative control (miR‐NC) were detected by qRT‐PCR and Western blot. E, The relative expression levels of miR‐20a‐5p in MDA‐MB‐231 cells transfected with miR‐20a‐5p inhibitor or negative control inhibitor (NC‐inhibitor). F, Transwell assay showed inhibition of miR‐20a‐5p suppressed migratory and invasive abilities of MDA‐MB‐231 cells. G and H, The expression levels of MMP‐2, MMP‐9, and TIMP‐3 in MDA‐MB‐231 cells transfected with miR‐20a‐5p inhibitor or negative control inhibitor (NC‐inhibitor) were detected by qRT‐PCR and Western blot. The data represent the mean ± SD from three independent experiments. **P < .01; ***P < .001. Student's *t* test

To determine whether the considerable expression of miR‐20a‐5p could affect the migration and invasion of breast cancer cells, miR‐20a‐5p or negative control miRNA (miR‐NC) were transfected into MDA‐MB‐231 cells. After confirmation of high expression of miR‐20a‐5p by RT‐PCR (Figure 2A, *P* < .001), Transwell assays were used to determine the effects of miR‐20a‐5p on breast cancer cell migration and invasion. Obviously, overexpression of miR‐20a‐5p in the MDA‐MB‐231 cells promoted the both migration and invasion of cells (Figure 2B, *P* < .01).

To further validate the effects of miR‐20a‐5p, the miR‐20a‐5p inhibitor or negative control inhibitor (NC‐inhibitor) was applied to lower the expression of miR‐20a‐5p. After verifying the depletion efficiency of miR‐20a‐5p by qRT‐PCR assay (Figure 2C, *P* < .01), the transwell assays were followed. The results showed that inhibitor of miR‐20a‐5p significantly inhibited the migration and invasion of MDA‐MB‐231 breast cancer cells (Figure 2D, *P* < .01).

Given the significant promotion of miR‐20a‐5p on the migration and invasion of MDA‐MB‐231 cells, we also detected the overexpression and inhibition of miR20a‐5p on the expression levels of MMP‐2, MMP‐9, and TIMP‐3. We observed that miR‐20a‐5p overexpression significantly upregulated the expression of MMP‐2, MMP‐9, while miR‐20a‐5p inhibitor downregulated the expression these two genes. As expected, miR‐20a‐5p overexpression significantly lowered the expression of TIMP3, which was upregulated by miR‐20a‐5p inhibitor (Figure 2C,D,G,H).

Similarly, overexpression of miR‐20a‐5p in MCF‐7 cells significantly promoted migration and invasion of MCF‐7 cells (Figure S1A‐D).

### 3.3 Breast cancer cell‐derived exosomes promoted the proliferation and differentiation of osteoclasts

**Figure 3:**
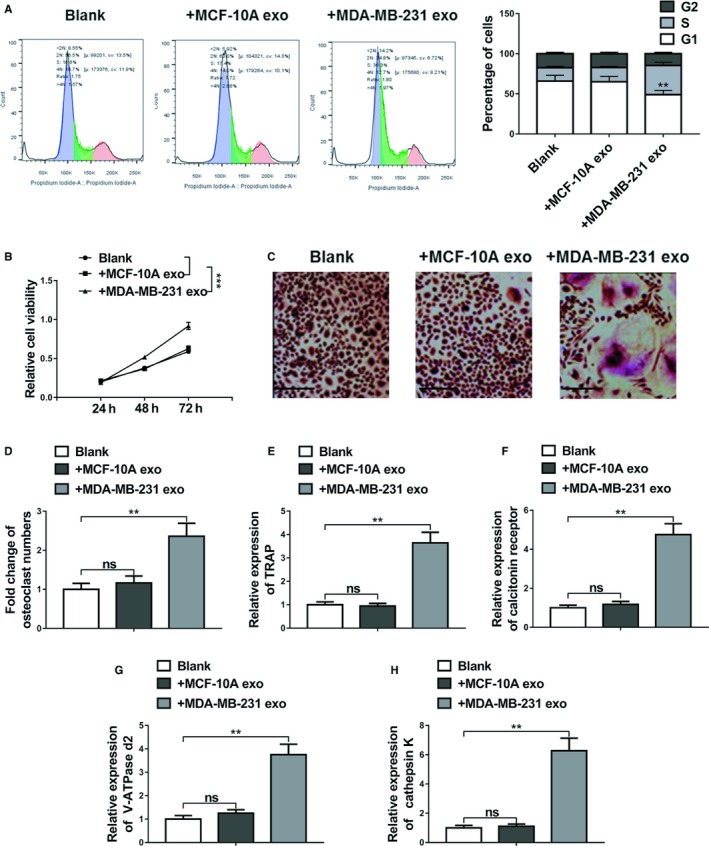
Breast cancer cell‐derived exosomes promoted the proliferation and differentiation of osteoclasts. A, Flow cytometric analyses of cell cycle distribution. Primary preosteoclasts were exposed to MCF‐10A cell‐derived exosomes (+MCF‐10A exo) or MDA‐MB‐231 cell‐derived exosomes (+MDA‐MB‐231 exo) or alone (blank) for 48 h. B, Cell viability was assessed by MTT assay following primary preosteoclasts were exposed to MCF‐10A cell‐derived exosomes (+MCF‐10A exo) or MDA‐MB‐231 cell‐derived exosomes (+MDA‐MB‐231 exo) or alone (blank) for 48 h. C and D, BMMs were cultured in osteoclastogenesis condition (M‐CSF+RNAKL) for 24 h after exposure to MCF‐10A cell‐derived exosomes (+MCF‐10A exo) or MDA‐MB‐231 cell‐derived exosomes (+MDA‐MB‐231 exo) or alone (blank) for 48 h, representative images of TRAP positive (pink or purple) BMMs were shown and number of TRAP‐positive osteoclasts in each well were counted, bar = 100 µm. E‐H, The relative mRNA expression of osteoclast differentiation marker genes including TRAP, calcitonin receptor, V‐ATPase d2, and cathepsin K was evaluated by qRT‐PCR after the same exposure. The data represent the mean ± SD from three independent experiments. *P < .05; **P < .01; ***P < .001 (two‐way ANOVA for B, Student's *t* test for others)

Primary preosteoclasts were exposed to MCF‐10A cell‐derived exosomes (+MCF‐10A exo) or MDA‐MB‐231 cell‐derived exosomes (+MDA‐MB‐231 exo) or alone (blank) for 48 hours. Flow cytometry was used to determine cell cycle composition. As shown in Figure 3A, the proportion of cells at the G1 phase was significantly lower in MDA‐MB‐231 cell‐derived exosomes group when compared to blank group. In comparison with MCF‐10A exo or blank groups, the proportion of cells at the S phase was remarkably elevated, while the proportion of cells at the G2 phases was significantly decreased in the MDA‐MB‐231 exo treated cells (*P* < .01).

The proliferation of primary osteoclasts was detected by MTT assays, and the results indicated that there was no significant difference for the cell proliferation of BMMs between the blank and MCF‐10A exo groups, whereas the cell proliferation activity in the MDA‐MB‐231 exo group was significantly increased (Figure 3B, *P* < .001).

BMMs were cultured in osteoclastogenesis condition (M‐CSF and receptor activator of RANKL) for 24 hours after exposure to MCF‐10A cell‐derived exosomes (+MCF‐10A exo) or MDA‐MB‐231 cell‐derived exosomes (+MDA‐MB‐231 exo) or alone (blank) for 48 hours, As demonstrated in Figure 3C, the quantity of TRAP‐positive multinucleated cells that counted as osteoclasts increased greatly in MDA‐MB‐231 exo group, suggesting the promoting effect of miR‐20a‐5p on osteoclast differentiation in BMMs. In addition, qRT‐PCR was performed to analyze the relative expression levels of osteoclast differentiation marker genes including TRAP, calcitonin receptor, V‐ATPase d2, and cathepsin K in BMMs. Data revealed that the relative expression of these markers in BMMs did not shown significant difference between the blank and MCF‐10A exo groups. However, the expression of these four differentiation markers were significantly upregulated in the MDA‐MB‐231 exo group, compared with the blank (Figure 3D‐H, *P* < .01).

### 3.4 MDA‐MB‐231 cell‐derived exosomes transferred miR‐20a‐5p to BMMs and facilitated the osteoclastogenesis

**Figure 4:**
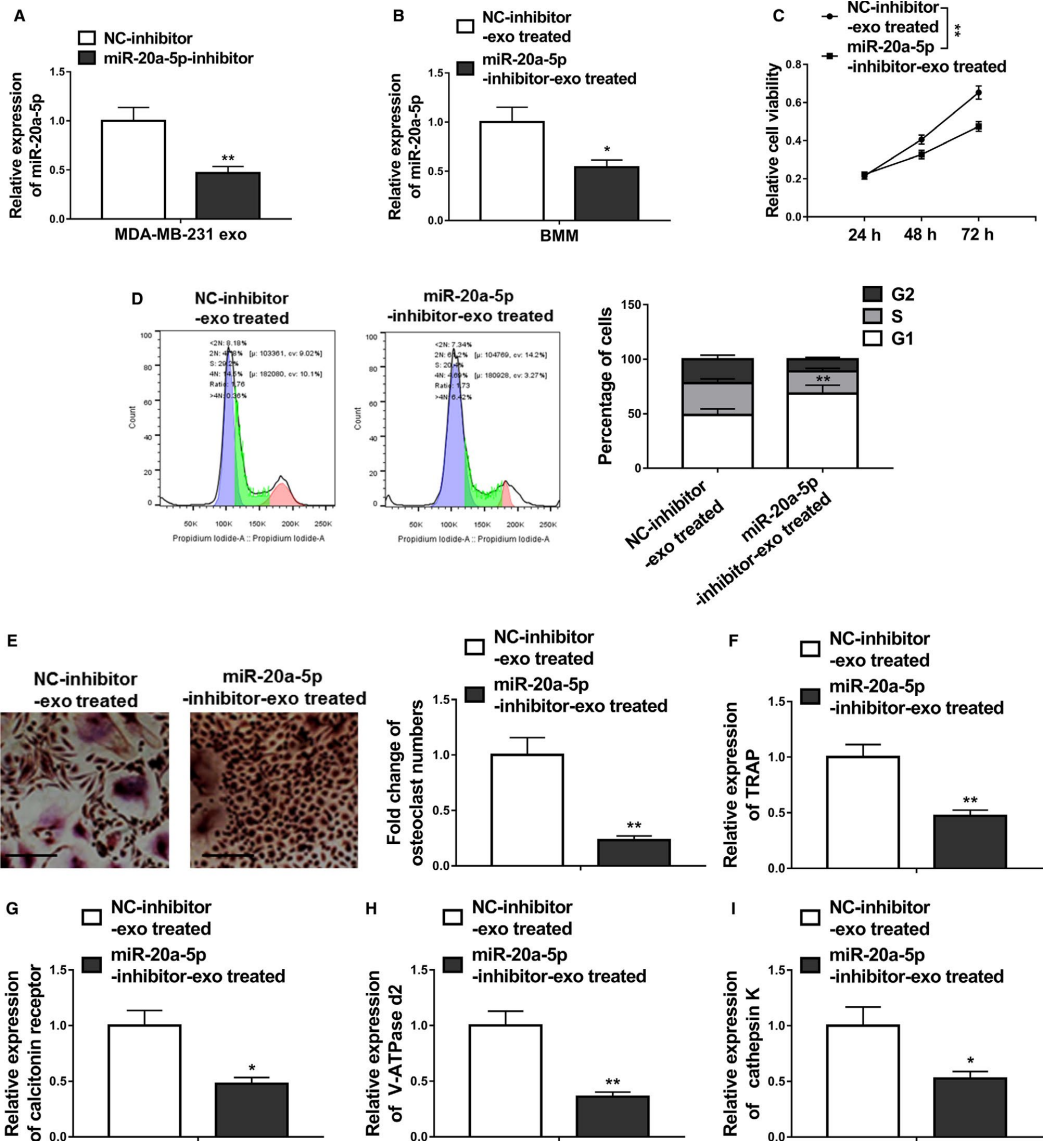
MDA‐MB‐231 cell‐derived exosomes transferred miR‐20a‐5p to BMMs and facilitated the osteoclastogenesis. A, qRT‐PCR revealing miR‐20a‐5p levels in MDA‐MB‐231 cell‐derived exosomes (MDA‐MB‐231 exo) transfected with miR‐20a‐5p inhibitor or negative control inhibitor (NC‐inhibitor). B, MiR‐20a‐5p levels in BMMs treated with MDA‐MB‐231 cell‐derived exosomes transfected with miR‐20a‐5p inhibitor (miR‐20a‐5p‐inhibitor‐exo treated) or negative control inhibitor (NC‐inhibitor‐exo treated) were measured by qRT‐PCR. C, Cell viability was assessed by MTT assay following primary preosteoclasts were exposed to MDA‐MB‐231 cell‐derived exosomes transfected with miR‐20a‐5p inhibitor (miR‐20a‐5p‐inhibitor‐exo treated) or negative control inhibitor (NC‐inhibitor‐exo treated). D, Flow cytometric analyses of cell cycle distribution. Primary preosteoclasts were exposed to MDA‐MB‐231 cell‐derived exosomes transfected with miR‐20a‐5p inhibitor (miR‐20a‐5p‐inhibitor‐exo treated) or negative control inhibitor (NC‐inhibitor‐exo treated). E, BMMs were cultured in osteoclastogenesis condition (M‐CSF+RNAKL) for 24 h after exposure to the same different MDA‐MB‐231 cell‐derived exosomes, representative images of TRAP positive (pink or purple) BMMs were shown and number of TRAP‐positive osteoclasts in each well were counted, bar = 100 µm. F‐I, The relative mRNA expression of osteoclast differentiation marker genes including TRAP, calcitonin receptor, V‐ATPase d2, and cathepsin K was evaluated by qRT‐PCR after the same exposure. The data represent the mean ± SD from three independent experiments. *P < .05; **P < .01 (two‐way ANOVA for C, Student's *t* test for others)

To verify the effects of miR‐20a‐5p from the exosomes of MDA‐MB‐231 on the osteoclastogenesis of BMMs, miR‐20a‐5p inhibitor or NC inhibitor were transfected into the BMMs incubated with MDA‐MB‐231 derived exosomes. RT‐PCR results showed that the relative expression of miR‐20a‐5p in the exosomes of MDA‐MB‐231 and BMMs were significantly downregulated by miR‐20a‐5p inhibitor (*P* < .01, Figure 4A,B). MiR‐20a‐5p inhibitor also inhibited the proliferation of BMMs (Figure 4C). In addition, the proportion of cells at the G1 phase in miR‐20a‐5p inhibitor treated cells was significantly higher than that in the NC‐inhibitor treated cells, whereas the proportion of cells at the G2 and S phases was decreased in the inhibitor group (Figure 4D).

The number of TRAP‐positive osteoclasts in the miR‐20a‐5p inhibitor group decreased significantly when compared with the NC‐inhibitor group (Figure 4E).

Furthermore, qRT‐PCR results demonstrated significantly downregulated levels of osteoclast differentiation marker genes including TRAP, calcitonin receptor, V‐ATPase d2, and cathepsin K in BMMs (Figure 4F‐I), indicating the inhibition of miR‐20a‐5p inhibitor on the osteoclastogenesis of BMMs. Interestingly, exosomes derived from miR‐20a‐5p‐overexpressing MCF‐7 cells greatly facilitated the osteoclastogenesis (Figure S2A‐I).

### 3.5 MiR‐20a‐5p promoted osteoclastogenesis by targeting SRCIN1

**Figure 5:**
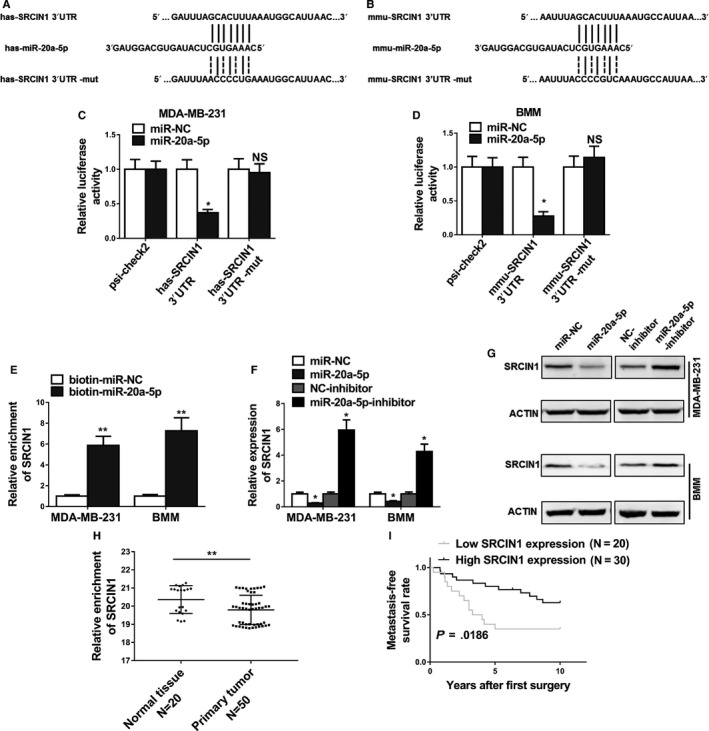
MiR‐20a‐5p promoted osteoclastogenesis by targeting SRCIN1. A and B, Predicted binding sites of miR‐20a‐5p in the wild type 3′UTR of SRCIN1 (SRCIN1 3′UTR) and mutations in the 3′UTR of SRCIN1 (SRCIN1 3′UTR‐mut) in humans and mice. C and D, The luciferase activities in MDA‐MB‐231 and BMMs cotransfected with indicated miR‐20a‐5p mimics or its negative control mimics (miR‐NC) and constructed luciferase reporter vectors (SRCIN1 3′UTR, SRCIN1 3′UTR‐mut, psi‐check2) were detected as the relative ratio of hRluc luciferase activity to hluc + luciferase activity. E, Detection of SRCIN1 mRNAs in biotinylated miRNA/target mRNA complex by real‐time RT‐PCR. The relative level of SRCIN1 mRNA in the complex pulled down by using biotinylated miR‐20a‐5p was compared to that of the complex pulled down by using the biotinylated control random RNA. F and G, The relative expression levels of SRCIN1 in MDA‐MB‐231 and BMMs cells transfected with indicated microRNA mimics or microRNA inhibitors detected by qRT‐PCR and Western blot. H, Expression levels of SRCIN1 in 20 normal tissues and 50 breast cancer tissues. I, Kaplan‐Meier's analysis of the correlation between SRCIN1 expression and the metastasis‐free survival of breast cancer patients. The data represent the mean ± SD from three independent experiments. *P < .05; **P < .01

**Figure 6:**
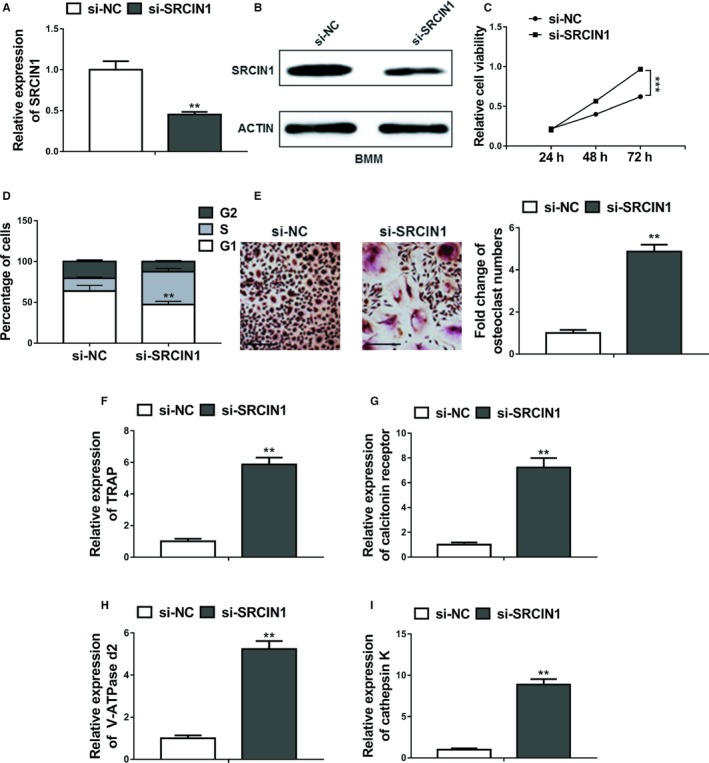
Knockdown of SRCIN1 promoted proliferation and differentiation of osteoclasts. A and B, The expression levels of SRCIN1 in BMMs transfected with SRCIN1 si‐RNA (si‐SRCIN1) or negative control si‐RNA (si‐NC) were measured by qRT‐PCR and Western blot. C, Cell viability was assessed by MTT assay following primary preosteoclasts were transfected with SRCIN1 si‐RNA (si‐SRCIN1) or negative control si‐RNA (si‐NC). D, Flow cytometric analyses of cell cycle distribution. Primary preosteoclasts were transfected with SRCIN1 si‐RNA (si‐SRCIN1) or negative control si‐RNA (si‐NC). E, BMMs were cultured in osteoclastogenesis condition (M‐CSF+RNAKL) for 24 h after transfection with SRCIN1 si‐RNA (si‐SRCIN1) or negative control si‐RNA (si‐NC), Representative images of TRAP positive (pink or purple) BMMs were shown and number of TRAP‐positive osteoclasts in each well were counted, bar = 100 µm. F‐I, The relative mRNA expression of osteoclast differentiation marker genes including TRAP, calcitonin receptor, V‐ATPase d2, and cathepsin K was evaluated by qRT‐PCR after transfection with SRCIN1 si‐RNA (si‐SRCIN1) or negative control si‐RNA (si‐NC). The data represent the mean ± SD from three independent experiments. **P < .01; ***P < .001 (two‐way ANOVA for C, Student's *t* test for others)

To decipher the mechanism of action of miR‐20a‐5p on osteoclastogenesis in breast cancer cells, we investigated the possible correlation between miR‐20a‐5p and potential targets. When we searched the targets of miR‐20a‐5p using bioinformatic tool Targetscan, SRCIN1 was included among the plentiful putative targets of miR‐20a‐5p (Figure S3). Further in silico analysis revealed that has‐SRCIN1 3′ UTR and mmu‐‐SRCIN13’ UTR contain the putative binding site of miR‐20a‐5p (Figure 5A,B). To validate the binding between miR‐20a‐5p and SRCIN1 3′ UTR, we performed the luciferase reporter assay using the WT or mutated SRCIN1 3′ UTR‐coupled luciferase reporter. As seen in Figure 5C and 5, the application of miR‐20a‐5p significantly downregulated the luciferase signal of WT SRCIN1 3′ UTR, in comparison with the miR‐NC (*P* < .05). These suppressive effects were abolished by mutated miR‐20a‐5p binding site of SRCIN1. Furthermore, Western blot results (Figure 5F,G) indicated that miR‐20a‐5p overexpressing significantly decreased the protein expression of endogenous SRCIN in MDA‐MB‐231 and BMM cells, the levels of which were significantly increased by the miR‐20a‐5p inhibitor when compared with NC‐inhibitor. The level of SRCIN was significantly lower in the primary tumor tissue samples than the one in the normal tissues from breast cancer patients (Figure 5H, *P* < .01). Accordingly, we found patients with high SRCIN expression showed significant higher metastasis‐free survival rate than patients with low SRCIN expression (Figure 5I). Furthermore, knockdown of SRCIN1 by si‐SRCIN1 significantly promoted proliferation and differentiation of osteoclasts compared to the negative control si‐RNA (si‐NC). First, qRT‐PCR and Western blot confirmed the downregulation of SRCIN1 in BMMs (Figure 6A,B). Furthermore, MTT assay showed that the relative cell viability in the si‐SRCIN1 transfected cells was significantly lower than the cells with si‐NC (Figure 6C), with more cells in S stage (Figure 6D). The number of osteoclasts in the si‐SRCIN1 group was significantly elevated (Figure 6E), suggesting the promoted proliferation of BMMs. The relative expression of osteoclast differentiation marker genes including TRAP, calcitonin receptor, V‐ATPase d2, and cathepsin K were significantly higher in the si‐SRCIN1 group when compared to the si‐NC group (Figure 6F‐I). All of these data suggested that SRCIN is a direct target of miR‐20a‐5p, while SRCIN and miR‐20a‐5p were negatively correlated in the pathogenesis of breast cancer.

## 4 DISCUSSION

Aggressive cancers are highly related to the robust biological interaction networks involving gene, miRNA, protein, as well as intracellular and intercellular cell interactions.^17^ Given the important participation of crosstalk between the bone microenvironment and tumor cells in the bone metastasis of breast cancer, and the well‐accepted role of exosomes in cell communication, we herein investigated one of the potential mechanisms of breast cancer cell derived exosomes on tumor cell invasion.

Previous studies have reported that various microRNAs participate in breast cancer progression and metastasis. These include miR‐9, −10b, −21, −29a, −155, −200a, −374a, and several other miRNAs.^14^ Notably, by detecting 20 normal mammary and 50 primary breast tumor tissue samples, we observed that the expression of mir‐20a‐5p in tumor tissue was significantly upregulated. To be noted, we used adjacent mammary tissue as normal control, because it is from same patients to minimize the interference of genetic background among different individuals. Although we tried to obtain the samples as far as possible from the tumor site, it is still possible to include some potential tumors unintentionally. Given this disadvantage, using samples from healthy volunteers as control could be more ideal in the future study. Coming back to the miRNA, exosomal miRNA in serum is considered as a potential marker for tumor diagnosis.^18^ Furthermore, specific exosomal miRNAs may modulate the tumor microenvironment. For instance, miR‐10b has been described as MDA‐MB‐231 cells derived exosomal miRNA that promotes cell invasion in human mammary epithelial (HMLE) cells.^14^ Consistently, in this study we found another specific miRNA, mir‐20a‐5p, was highly expressed in the cells and exosomes of MDA‐MB‐231 cells. Interestingly, the expression of mir‐20a‐5p in MDA‐MB‐231 cells was significantly higher than those in other detected cell types including primary murine BMMs, MCF‐10A, and MCF‐7. We have to mention that the selection of the cell model is a limitation of this present study. Specifically, although the patient specimens in our study involved different subtypes of breast cancer, in which high expression of MDA‐MB‐231 was observed, we focused on the MDA‐MB‐231 cells as a model for more thorough exploration. While MCF7 is estrogen, progesterone receptors ^+^, HER2^−^, MDA‐MB‐231 is a triple‐negative breast cancer cell line. Correspondingly, effective designed hormone therapy strategy has been designed for patients with positive hormone receptor or HER2^+^, whereas chemotherapy is the only systemic therapy for TNBC patients.^19^ It has been reported that miR‐20a‐5p was highly expressed in both TNBC tissues and cell lines, and the overexpression of miR‐20a‐5p promoted the migration and invasion of TNBC cells in vitro.^18^ In line with this, our Transwell assay results showed that overexpression of miR‐20a‐5p promoted migratory and invasive abilities of MDA‐MB‐231 cells, which were suppressed significantly by the miR‐20a‐5p inhibitor, indicating that miR‐20a‐5p is a breast cancer cell invasion promoter. In the future, we would also use in vivo animal model to validate these results.

To further investigate the promoting role of miR‐20a‐5p on osteoclasts, primary pre‐osteoclasts BMMS were incubated with MCF‐10A and MDA‐MB‐231 cell‐derived exosomes. The results showed that MDA‐MB‐231 exosomes promoted the cell cycle progression and proliferation of osteoclasts. Furthermore, the relative expression levels of osteoclast differentiation marker genes including TRAP, calcitonin receptor, V‐ATPase d2, and cathepsin K in BMMs were significantly upregulated by MDA‐MB‐231 exosomes exposure. Furthermore, all of the promotion could be inhibited by the administration of miR‐20a‐5p inhibitor, suggesting that miR‐20a‐5p was the key component responsible for the promotion of MDA‐MB‐231 cell‐derived exosomes on the proliferation and differentiation of osteoclasts.

This present study we have demonstrated that miR‐20a‐5p level in MDA‐MB‐231 cell‐derived exosomes could be inhibited by miR‐20a‐5p inhibitor, and this level can be passed to the cultured BMMs. MiR‐20a‐5p levels are dysregulated in various human cancers. Specifically, miRNA‐20a‐5p has been reported to elevate and promote colorectal cancer invasion and metastasis,^20^ but remained the same as normal control in atrophic gastritis and gastric cancer.^21^ Enhanced miRNA‐20a‐5p was observed in triple‐negative breast tumors than that in luminal A ones,^22^ which was confirmed in our study. Recent studies have shown that miRNAs exert considerable roles in regulating various biological processes of osteoblast and osteoclast differentiation and function, thus making miRNAs as biomarkers and potential targets for osteoporosis therapy.^23^ Generally, miRNAs with upregulated expression level during osteoclast differentiation or formation tend to promote osteoclastogenesis, while miRNAs with downregulated level during osteoclast differentiation tend to inhibit osteoclastogenesis.^24^ A previous study using micro array analysis revealed the expression profile during different stages of murine osteoclastogenesis. Among all detected miRNAs, 49 were upregulated and 44 were downregulated,^25^ however, miR‐20a‐5p was not among the list. Therefore, the modulation of miR‐20a‐5p could not be explained by the direct regulation on osteoporosis, and we were fascinated to explore its mechanism of actions.

In silico analysis revealed that the 3′ UTRs of both human and murine SRCIN1 contain the putative binding site of miR‐20a‐5p. SRCIN1, the SRC kinase signaling inhibitor 1, plays important roles in inactivating SRC and thus suppress tumor in cancers via different mechanisms.^26^ For example, previous report demonstrated that SRCIN1 inhibited proliferation and invasion of cancer cells by regulating the SRC or E‐cadherin/EGFR signaling pathways.^27^ Mutually, there was study presenting that miR‐150 represses SRCIN1 translation in lung cancer.^28^ Researchers also showed that expression of SRCIN1 was significantly lower in the osteosarcoma cell lines than in osteoblastic cell line.^26^ Furthermore, the expression level of SRCIN1 was negatively correlated with tumor malignancy in breast cancer and SRCIN1 inhibited the invasion of metastatic breast carcinoma cells.^29^ In our study we confirmed the downregulated expression of SRCIN1 in primary tumors than normal tissue, and showed that miR‐20a‐5p overexpressing significantly decreased the protein expression of endogenous SRCIN in MDA‐MB‐231 and BMM cells, the levels of which were significantly increased by the miR‐20a‐5p inhibitor when compared with NC‐inhibitor. All of these results indicated that SRCIN is a direct target of miR‐20a‐5p, while SRCIN and miR‐20a‐5p were negatively correlated in the pathogenesis of breast cancer. To be noted, miR‐20a has been reported to regulate adipocyte differentiation by targeting TGF‐β signaling,^30^ which is important growth factor in tumor microenvironment as earlier discussed, making miR‐20a‐5p as a more potential target for breast cancer therapy.

## 5 CONCLUSION

In conclusion, the present work provides evidence that miR‐20a‐5p transferred from breast cancer cell‐derived exosomes promotes the proliferation and differentiation of osteoclasts by targeting SRCIN1, providing scientific foundations for the development of exosome or miR‐20a‐5p targeted therapeutic intervention in breast cancer progression. To further confirm their participation in bone metastasis, an in vivo study is warranted, which is a limitation in the current study.

## CONFLICT OF INTEREST

All the authors declare that they have no conflict of interest.

## Data Availability

Data could be obtained to the corresponding author upon reasonable request.
