# Supplementary material for: Breast cancer cell‐derived exosomal miR‐20a‐5p promotes the proliferation and differentiation of osteoclasts by targeting SRCIN1
Source: Cancer Med. 2019 Aug 6;8(12):5687–701. doi: 10.1002/cam4.2454 (PMC6745844; doi:10.1002/cam4.2454)

**Supplementary Materials**

**Table S1.** Correlation of miR-20a-5p expression with clinicopathological features in 50 breast cancer patients

| Expression of miR-20a-5p | | | | |
| --- | --- | --- | --- | --- |
| Variables Low (%) High (%) P value | | | | |
| Age |  | |  | 0.7733 |
| <55 | | 9(36.0%) | 16(64.0%) |  |
| ≥55 | | 11(44.0%) | 14(56.0%) |  |
| TNM staging | |  |  | 0.0352 |
| Ⅰ | | 11(61.1%) | 7(38.9%) |  |
| Ⅱ-Ⅳ | | 9(28.1%) | 23(71.9%) |  |
| Tumor size (cm) | |  |  | 0.7654 |
| <5 | | 8(44.4%) | 10(55.6%) |  |
| ≥5 | | 12(37.5%) | 20(62.5%) |  |
| Bone metastasis | |  |  | 0.0084 |
| Yes | | 4(18.2%) | 18(81.8%) |  |
| no | | 16(57.1%) | 12(42.9%) |  |
| ER status | |  |  | 0.5691 |
| Positive | | 8(34.8%) | 15(65.2%) |  |
| Negative | | 12(44.4%) | 15(55.6%) |  |
| PR status | |  |  | 0.7739 |
| Positive | | 10(37.0%) | 17(63.0%) |  |
| Negative | | 10(43.5%) | 13(56.5%) |  |
| HER2 status | |  |  | 0.7737 |
| Positive | | 13(41.9%) | 18(58.1%) |  |
| Negative | | 7(36.8%) | 12(63.2%) |  |


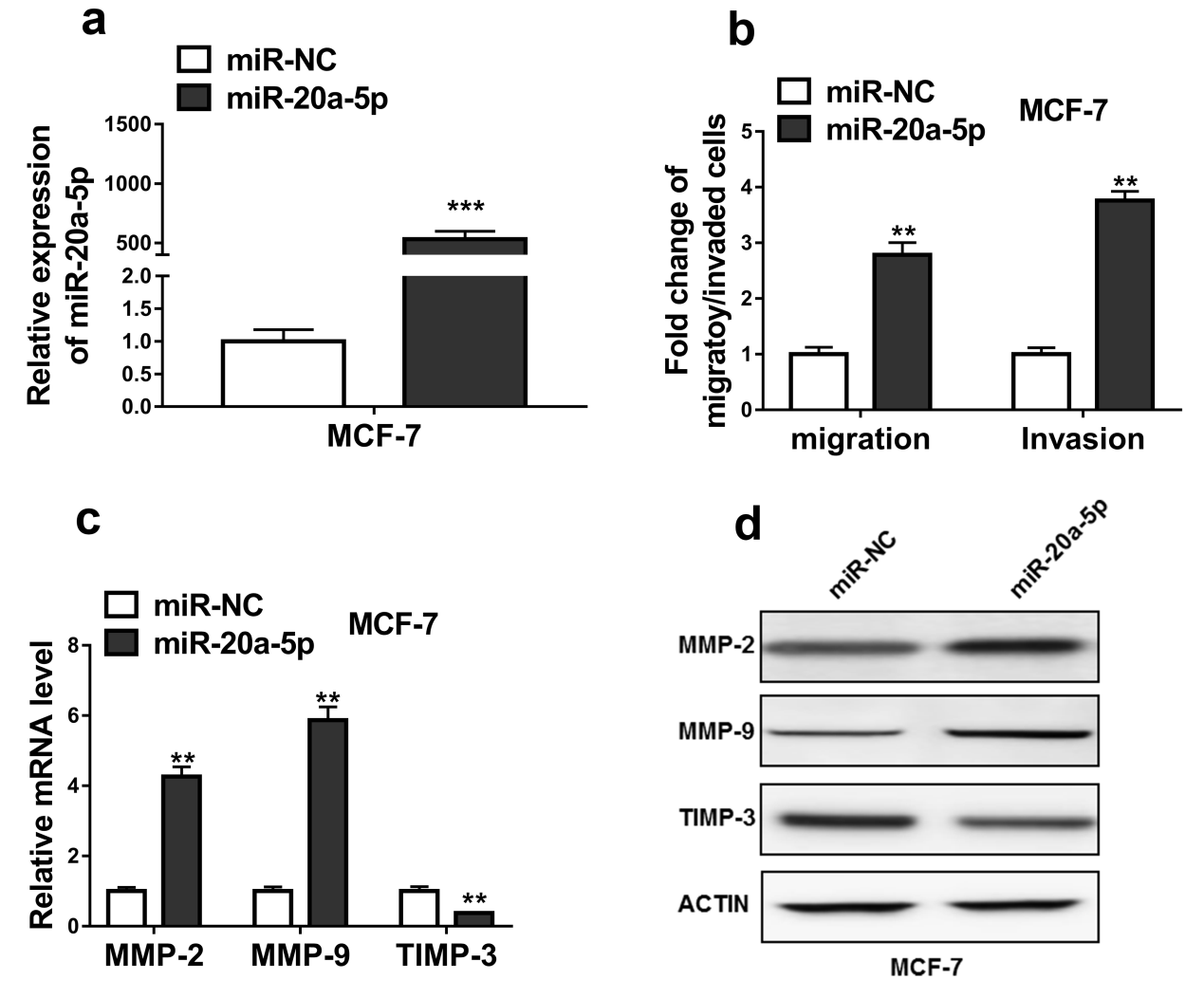
**Figure S1**. Overexpression of miR-20a-5p significantly promoted migration and invasion of MCF-7 cells. (a) The relative expression levels of miR-20a-5p in MCF-7 cells transfected with miR-20a-5p mimics or miRNA negative control (miR-NC). (b) Transwell assay showed overexpression of miR-20a-5p promoted migratory and invasive abilities of MCF-7 cells. (c and d) The expression levels of MMP-2, MMP-9 and TIMP-3 in MCF-7 cells transfected with miR-20a-5p mimics or miRNA negative control (miR-NC) were detected by qRT-PCR and western blot. The data represent the mean ± SD from three independent experiments. *p<0.05; **p<0.01; *** p<0.001. Student’s t-test.


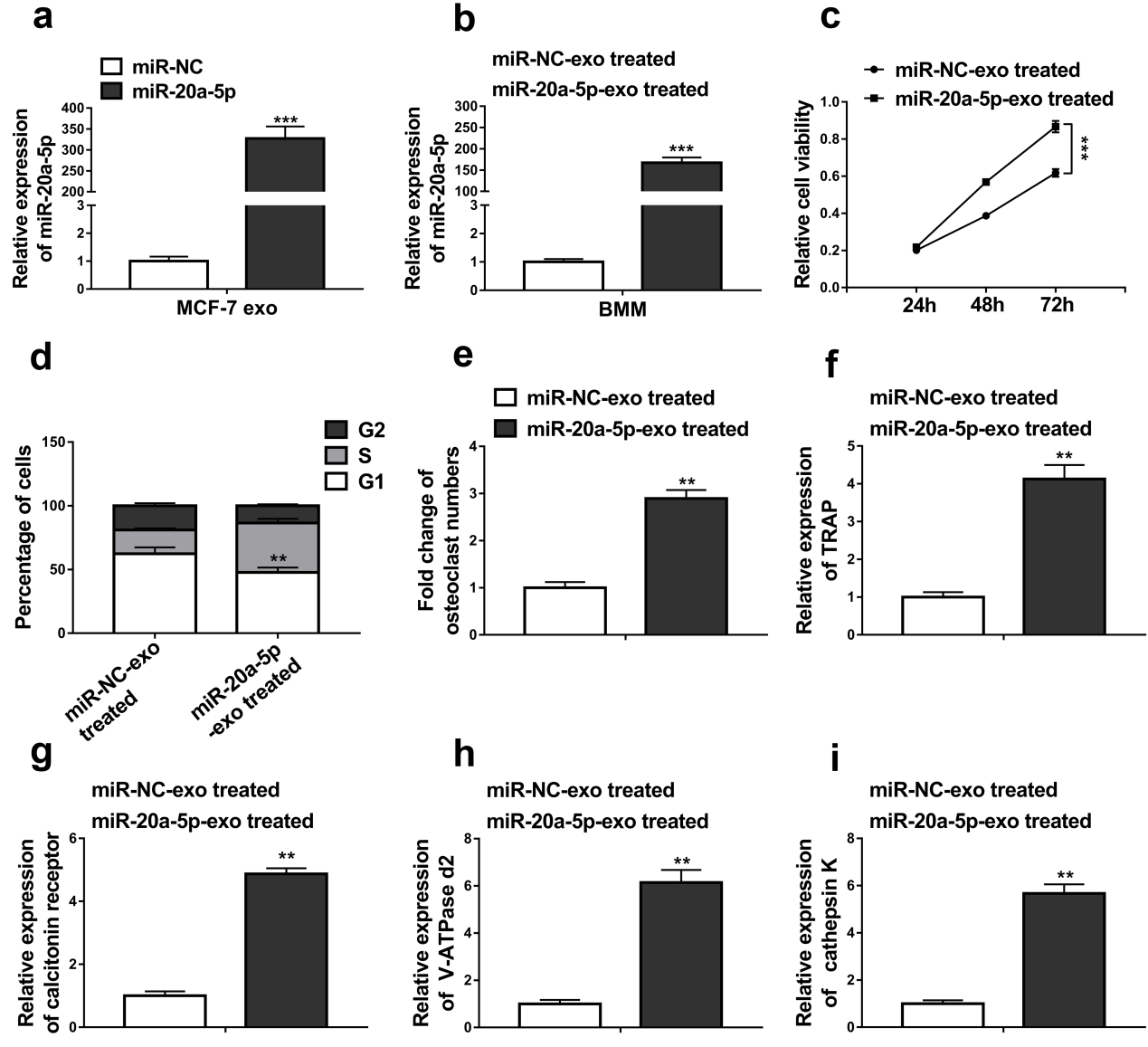
**Figure S2**. Exosomes derived from miR-20a-5p-overexpressing MCF-7 cells facilitated the osteoclastogenesis. (a) qRT-PCR revealing miR-20a-5p levels in MCF-7 cell-derived exosomes (MCF-7 exo) transfected with miR-20a-5p mimics or miRNA negative control (miR-NC). (b) MiR-20a-5p levels in BMMs treated with MCF-7 cell-derived exosomes transfected with miR-20a-5p mimics (miR-20a-5p-exo treated) or miRNA negative control (miR-NC-exo treated) were measured by qRT-PCR. (c) Cell viability was assessed by MTT assay following primary preosteoclasts were exposed to MCF-7 cell-derived exosomes transfected with miR-20a-5p mimics (miR-20a-5p-exo treated) or miRNA negative control (miR-NC-exo treated). (d) Flow cytometric analyses of cell cycle distribution. Primary preosteoclasts were exposed to MCF-7 cell-derived exosomes transfected with miR-20a-5p mimics (miR-20a-5p-exo treated) or miRNA negative control (miR-NC-exo treated). (e) BMMs were cultured in osteoclastogenesis condition (M-CSF+ RNAKL) for 24 h after exposure to the same different MCF-7 cell-derived exosomes, number of TRAP-positive osteoclasts in each well were counted. (f-i) The relative mRNA expression of osteoclast differentiation marker genes including TRAP, calcitonin receptor, V-ATPase d2, and cathepsin K was evaluated by qRT-PCR after the same exposure. The data represent the mean ± SD from three independent experiments. *p<0.05; **p<0.01 (two-way ANOVA for c, student’s t-test for others).

**Figure S3.** Some of the putative targets of miR-20a-5p predicted in Targetscan（http://www.targetscan.org/vert_71/）.


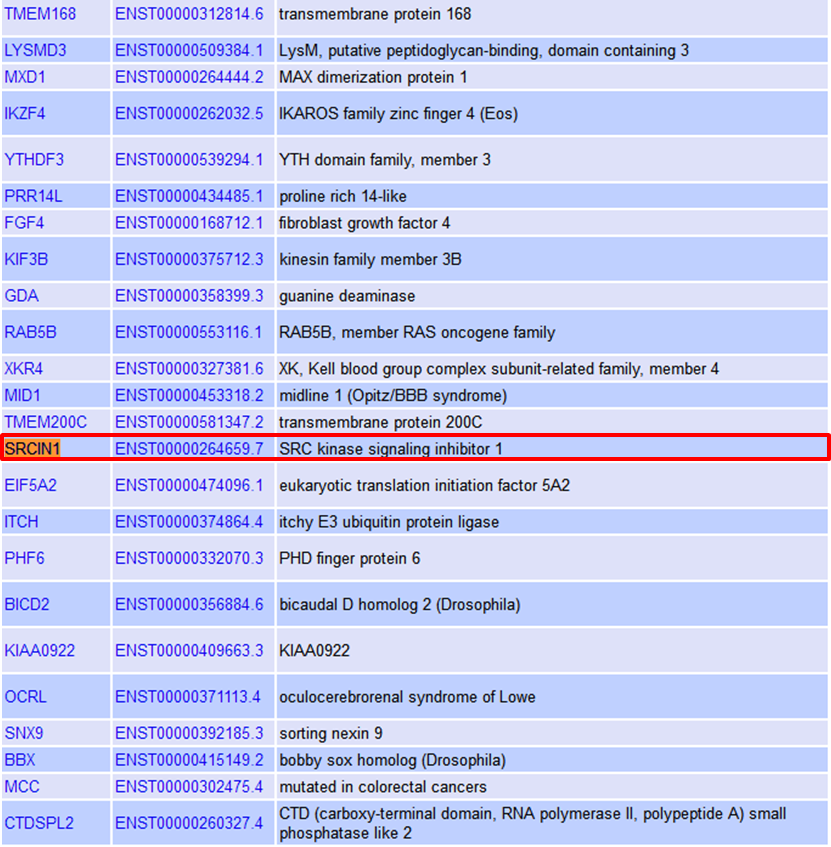

Supplement: Supplementary file 1 [file CAM4-8-5687-s001.docx]
